# Multispectral versus texture features from ZiYuan-3 for recognizing on deciduous tree species with cloud and SVM models

**DOI:** 10.1038/s41598-023-28532-0

**Published:** 2023-05-05

**Authors:** Xiao Liu, Ling Wang, Xiaolu Liu, Langping Li, Xicun Zhu, Chunyan Chang, Hengxing Lan

**Affiliations:** 1 State Key Laboratory of Resources and Environmental Information System, Institute of Geographic Sciences and Natural Resources Research, Chinese Academy of Sciences, Beijing, 100101 China; 2 University of Chinese Academy of Sciences, Beijing, 100049 China; 3 College of Resources and Environment, Shandong Agricultural University, Tai’an, 271018 China; 4 National Engineering Laboratory for Efficient Utilization of Soil and Fertilizer Resources, Shandong Agricultural University, Tai’an, 271018 China; 5 School of Geological Engineering and Geomatics, Chang’an University, Xi’an, 710064 China; 6 Key Laboratory of Ecological Geology and Disaster Prevention of Ministry of Natural Resources, Xi’an, 710054 China

**Keywords:** Forest ecology, Forestry

## Abstract

Tree species recognition accuracy greatly affects forest remote sensing mapping and forestry resource monitoring. The multispectral and texture features of the remote sensing images from the ZiYuan-3 (ZY-3) satellite at two phenological phases of autumn and winter (September 29th and December 7th) were selected for constructing and optimizing sensitive spectral indices and texture indices. Multidimensional cloud model and support vector machine (SVM) model were constructed by the screened spectral and texture indices for remote sensing recognition of *Quercus acutissima* (*Q. acutissima*) and *Robinia pseudoacacia* (*R. pseudoacacia*) on Mount Tai. The results showed that, the correlation intensities of the constructed spectral indices with tree species were preferable in winter than in autumn. The spectral indices constructed by band 4 showed the superior correlation compared with other bands, both in the autumn and winter time phases. The optimal sensitive texture indices for both phases were mean, homogeneity and contrast for *Q. acutissima*, and contrast, dissimilarity and second moment for *R. pseudoacacia.* Spectral features were found to have a higher recognition accuracy than textural features for recognizing on both *Q. acutissima* and *R. pseudoacacia,* and winter showing superior recognition accuracy than autumn, especially for *Q. acutissima.* The recognition accuracy of the multidimensional cloud model (89.98%) does not show a superior advantage over the one-dimensional cloud model (90.57%). The highest recognition accuracy derived from a three-dimensional SVM was 84.86%, which was lower than the cloud model (89.98%) in the same dimension. This study is expected to provide technical support for the precise recognition and forestry management on Mount Tai.

## Introduction

Forest resource monitoring is an essential requirement for protecting the forestry environment and promoting forestry development^1–3^. Mount Tai is a typical forest mountain widely distributed deciduous broad-leaf tree species, which located in a warm temperate zone and named as a famous tourist scenery in China. Mount Tai has a diversity of tree species, and the proportion of woodland area exceeds 80% of the total vegetation area^4^. However, the complex terrain, inconvenient transportation and difficulty performing field investigations lead to the difficulty for recognizing a large spatial scale investigation over a short time^5^, which affects the dynamic monitoring of forestry resource information at Mount Tai. Remote sensing technology has the characteristics of large coverage, short cycles and repeatability, which results in advantages over the recognition of forest tree species^6^. Hyperspectral remote sensing has the characteristics of high dimensionality, high correlation among bands and spectral mixing which would easily cause redundancy^7^. Multispectral remote sensing has the characteristics of faster data acquisition, lower cost and longer timeliness, especially in high resolution images, which could elaborate on the details of tree species^8,9^. The relative completeness and consistency of spectral and geometric information in space and time strongly facilitates the rapid recognition of tree species and large-scale mapping^10–12^.

Spectral and spatial texture features have been widely applied in remote sensing recognition, and numerous research findings have been obtained from it^13–18^. Existing tree recognition algorithms include support vector machine, artificial neural networks (ANNs), the minimum distance method, the maximum likelihood classification (MLC), and the decision tree classification method^18,19^. Researchers have evaluated the recognition performance of these methods comparing. For example, the improved MLC and ANNs were applied to multi-temporal Indian remote sensing satellite (IRS)-1B images to classify wheat crops in two areas of India, and the results of the ANN classification were superior to those of the MLC^20^. The airborne detection of tree species was accomplished using the two-stage SVM classifier to first predict tree species in Kruger National Park from hyperspectral data at the pixel scale and then combine the tree crown-level information with the pixel-level species probabilities, which resulted in an overall prediction accuracy of 76% for 15 species^21^. The decision tree classification method was used to recognize rubber plantations along the border region of China, Laos and Myanmar based on the spectral features and texture characteristics of Landsat remote sensing image data and Moderate Resolution Imaging Spectroradiometer-normalized difference vegetation index (MODIS-NDVI) data, respectively, and the overall accuracy of the mature rubber forest exceeded 90%, and that of the young rubber forest exceeded 75%^22^. Data from the airborne light detection and ranging canopy height model and the hyperspectral compact airborne spectrographic imager were combined to take advantage of the vertical structural and spectral information to identify birch, red pine, larch and spruce-fir in a natural temperate forest in the Liangshui National Nature Reserve (Heilongjiang Province in northeastern China) based on SVM, which successfully identified the tree species, with an overall accuracy of 83.88%^23^. Shen X et al.^24^ used the integrated sensor Li CHy (which integrated LiDAR, charge-coupled device (CCD) and hyperspectral sensors) to obtain both high-resolution imagery and hyperspectral data at the same time for the natural secondary forest in the southern Jiangsu hilly region of China, and the tree species and forest types were classified using the back propagation (BP) neural network. The overall accuracy was 64.6% for the four typical tree species (*Pinus massoniana*, *Quercus acutissima*, *Castanea mollissima* and *Liquidambar formosana*) and 81.1% for forest types. Multi-source and multi-temporal remote sensing data (Landsat 8 operational land imager (OLI) in winter and Worldview-2 in summer), combined with expert knowledge and phenological characteristics of the tree species, were used to identify species in Shennongjia, and the accuracy was 70.18% for evergreen *Quercus spinosa* and 65.21% for deciduous tree species^25^. In summary, the minimum distance and maximum likelihood methods were the traditional algorithms in remote sensing recognition. These methods had the characteristics of mature application, simple implementation, a small amount of computation and fast speeds, but the disadvantage was that the recognition accuracy was insufficient, and only the samples with obvious features could be correctly recognized^26^. Studies have shown that SVM have been widely used for the accurate recognition of forest species and shown higher recognition performance than other methods^26,27^.

Cloud models are an effective tool for converting qualitative concepts into quantitative formulations and are capable of capturing fuzzy and stochastic uncertainty with simple numerical features, i.e., *Expect* (*Ex*), *Entropy* (*En*), and *Hyper-Entropy* (*He*). This capturing of uncertainty is achieved by introducing probability theory into the fuzzy set framework and obtaining membership degrees using probability density functions. Clouds are a new and readily visualizable concept of uncertainty. Clouds are combined with several cloud drops, in which the cloud shape reflects the most essential features of the quantitative concept. Cloud model is operationally simple, and extensive model computation is unnecessary^19^. In recent years, the cloud model theory has been applied to the field of data mining, such as remote sensing image interpretation. Du et al.^28^ proposed the concept of the cloud transformation partition method for the cloud model, which also proved the feasibility of the cloud model in remote sensing image recognition. There are already plenty of research findings for image segmentation based on the cloud model^19,29–31^. Cloud model has also been applied to identify emitters, which been proved the applicability and validity of cloud model recognition^32^. However, the cloud model is currently still uncommonly applied in tree species recognition, and the model performance for tree species recognition deserves to be profoundly explored.

In this study, the spectral and texture features from a multispectral remote sensing image via the ZiYuan-3 (ZY-3) satellite were put into one-dimensional or multi-dimensional cloud model for the remote sensing recognition of the most dominant deciduous broad-leaved tree species of Mount Tai, *Q. acutissima* and *R. pseudoacacia*^33,34^. Meanwhile, the SVM models were constructed for comparison. We aim to improve the recognition accuracy of deciduous broad-leaves tree species on Mount Tai and provide a more convenient and feasible method for tree recognition.

## Data and methods

### Research area

**Figure 1:**
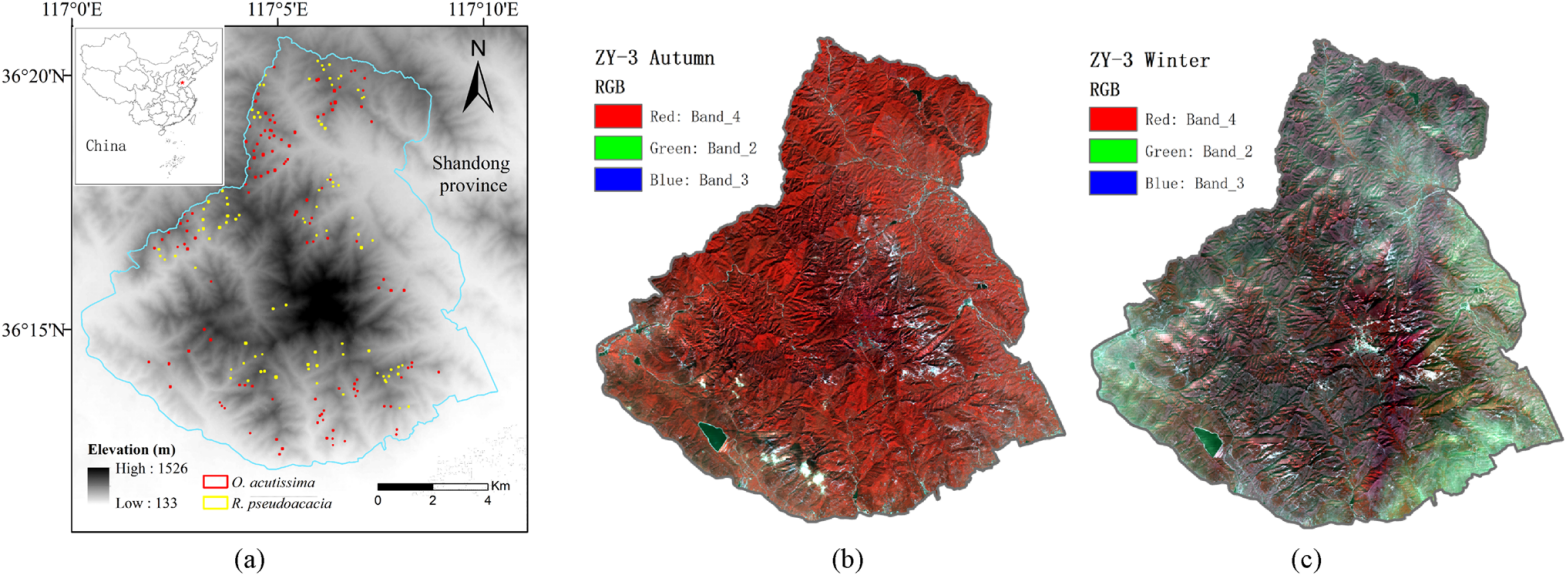
Spatial distribution of samples and multispectral images. (**a**) Location map of the study region and distribution of the *Q. acutissima* and *R. pseudoacacia* samples. (**b**) Autumn image on September 29, 2014; (**c**) Winter image on December 7, 2014. The map was generated using “Esri ArcMap (10.6.0.8321)” package (https://www.esri.com/en-us/arcgis/products/arcgis-desktop/overview).

Mount Tai (116°50′-117°12′E, 36°11′-36°31′N) is located in the centre of Shandong Province and has a warm temperate continental monsoon climate (Fig. 1a). The seasonal features around the mountain are extremely distinguishable. Vertical zonation features result in different mean annual temperatures between the summit and the foot of the mountain, 5.3◦ C and 12.8◦ C, respectively. Annual precipitation increases gradually with altitude, about 722.6 mm at the foot of the mountain and 1132 mm at the top. The soil is predominantly slightly acid brown soil with a thin layer of about 20–30 cm. The vegetation cover is over 90%, with forest cover of about 81.5%. Deciduous broad-leaved forests are the main vegetation in Mount Tai, dominated by planted forests and natural secondary forests and with the largest proportion of pure forests. There are *Q. acutissima, R. pseudoacacia, Pinus tabulaeformis, Platycladus orientalis, Larch,* and *Bamboo*. In which, *Q. acutissima* and *R. pseudoacacia* are the largest deciduous broad-leaf tree species on Mount Tai^33^.

### Multispectral data

Multispectral remote sensing images from ZY-3 on September 29 (Fig. 1b) and December 7 (Fig. 1c), 2014, were selected to recognize the tree species, considering the phenological characteristics of tree species, the satellite return cycle and weather factors. Mount Tai images from ZY-3 in 2014 include four scenes on June 13, September 29, October 9 and December 7. The vegetation was flourishing, with a criss-cross pattern, and easy to misclassify in June. *Q. acutissima* and *R. pseudoacacia* are changing leaf colour in September, and the shape and colour of the leaves were different and easier to identify. The beginning of December is the defoliating period of the deciduous broad-leaf forest, which is easily distinguished from the coniferous forest. The differences in the fresh leaf and canopy characteristics between the two tree species were larger and easier to identify than those in other phases. Moreover, by taking into account the image quality and closeness of the September 29 and October 9 dates, images from ZY-3 on September 29 (Fig. 1b) and December 7 (Fig. 1c), 2014, were finally selected. The images have 4 bands, with spectral ranges of 0.45–0.52 µm, 0.52–0.59 µm, 0.63–0.69 µm and 0.77–0.89 µm, and the spatial resolution is 5.8 m.

Sample area selection in the plant species survey followed the principle of uniformity and typicality of tree species distribution. For example, the tree sampling plots were uniformly distributed in three elevation zones of 400–600 m, 600–800 m, and 800–1000 m. Based on the elevational distribution features of *Q. acutissima* and *R. pseudoacacia* in Mount Tai, the proportion of sample numbers in these three elevation zones followed the formula of 1:3:2. Each tree species sample plot are far from cloud cover and slope tops, large stream gullies and rocky outcrops, as shown in Fig. 1a. In addition, pixel differences could not be discounted and have been selected as experimental units. There were 2550 pixels in the *Q. acutissima* area and 1635 pixels in the *R. pseudoacacia* area. Equidistant sampling was applied to select 2/3 pixels as modelling samples (1700 of *Q. acutissima* and 1104 of *R. pseudoacacia*) and 1/3 pixels as validation samples (850 of *Q. acutissima* and 552 of *R. pseudoacacia*) for each tree species.

### Research methods

**Figure 2:**
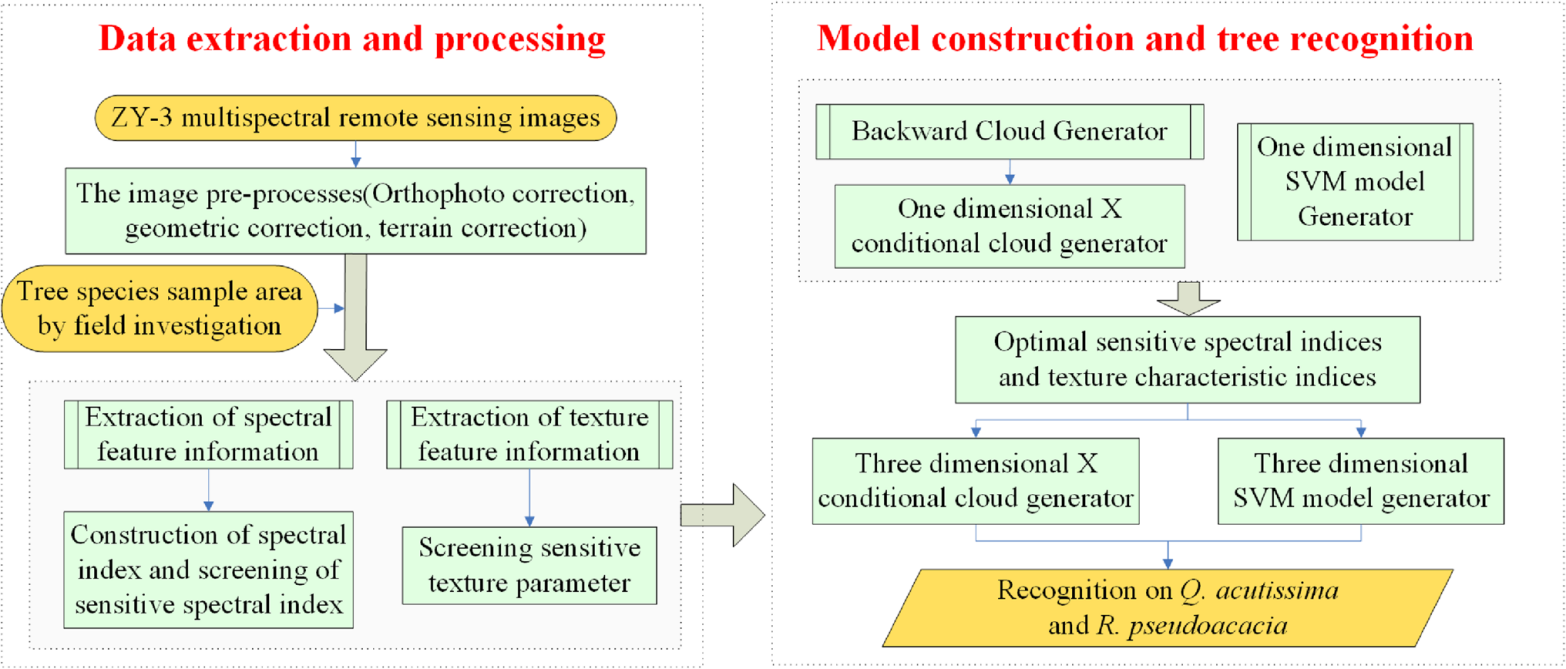
Flowchart of the tree species recognition technology.

The pre-processing for ZY-3 multispectral remote–sensing images, such as atmospheric correction, geometrical correction and radiometric correction, had been performed before the species recognition. Topographic radiometric correction was specifically verified multiple times to reduce the influence of the complex topography of Mount Tai on the recognition results. The model construction and tree identification technology flowchart are shown in Fig. 2.

#### Spectral indices

Mathematical algorithms were applied to construct the spectral indices based on the pixel reflectance of the multispectral images, the categories of 22 spectral indices and the corresponding mathematical formulations are shown as Table 1. A total of 166 spectral indices were constructed at each image phase (2014 September 29 and December 7) based on the four bands^35^. The logistic regression model was applied to the correlation analysis between the spectral indices and tree species, in which the spectral indices and tree species types were quantitative independent variables and qualitative dependent variables, respectively. The spectral indices with the highest correlation coefficients were determined to be sensitive spectral indices, which is used as the conditional attributes to construct one-dimensional or multi-dimensional cloud models and SVM models. Then, the three optimal sensitive spectral index was selected according to the recognition accuracy of the one-dimensional model to construct the three–dimensional models.

**Table 1:** Construction formulations of spectral indices.

Single band		Multi-band			
*F*_*1*_ (*i*)	*R*_*i*_	*F*_*8*_ (*i, j*)	*R*_*i*_ + *R*_*j*_	*F*_*16*_ (*i, j*)	(*R*_*i*_ − *R*_*j*_)*/*(*R*_*i*_*R*_*j*_)
*F*_*2*_ (*i*)	*R*_*i*_^*2*^	*F*_*9*_ (*i, j*)	*R*_*i*_ − *R*_*j*_	*F*_*17*_ (*i, j*)	(*R*_*i*_*/R*_*j*_)*/*(*R*_*i*_ − *R*_*j*_)
*F*_*3*_ (*i*)	*R*_*i*_^*3*^	*F*_*10*_ (*i, j*)	*R*_*i*_*R*_*j*_	*F*_*18*_ (*i, j*)	(*R*_*i*+_*R*_*j*_)*/e*^*Ri*^
*F*_*4*_ (*i*)	*R*_*i*_^*0.5*^	*F*_*11*_ (*i, j*)	*R*_*i*_*/R*_*j*_	*F*_*19*_ (*i, j*)	(*R*_*i*_ − *R*_*j*_)*/e*^*Ri*^
*F*_*5*_ (*i*)	*R*_*i*_^*1/3*^	*F*_*12*_ (*i, j*)	*lnR*_*i*_*/*(*R*_*i*_ − *R*_*j*_)	*F*_*20*_ (*i, j*)	*lnR*_*i*_*/*(*R*_*i*_ − *R*_*j*_)
*F*_*6*_ (*i*)	*e*^*Ri*^	*F*_*13*_ (*i, j*)	(*R*_*i*_ − *R*_*j*_)*/*(*R*_*i*_ + *R*_*j*_)	*F*_*21*_ (*i, j*)	*lnR*_*i*_*/*(*R*_*i*_ + *R*_*j*_)
*F*_*7*_ (*i*)	*lnR*_*i*_	*F*_*14*_ (*i, j*)	(*R*_*i*_*R*_*j*_)*/*(*R*_*i*_ + *R*_*j*_)	*F*_*22*_ (*i, j*)	*lnR*_*i*_*/*(*R*_*i*_ − *R*_*j*_)
		*F*_*15*_ (*i, j*)	(*R*_*i*_*/R*_*j*_)*/*(*R*_*i*_ + *R*_*j*_)		

*F*_*1*_* − F*_*22*_ represents the categories of 22 spectral indices constructed by different mathematical calculation methods. *R*_*i*_, *R*_*j*_ (*i*, *j* = 1, 2, 3, 4) represents the band reflectance of the images.

#### Texture feature parameters

The grey level co-occurrence matrix is an algorithm proposed by Haralick et al.^36^ to describe the texture features. This matrix is used to reflect the grey relation of pixels in a certain area and the distance in terms of direction, adjacent spacing, and amplitude of variation, which represents the spatial correlation of the greyscale in the image. There are 14 kinds of texture feature parameters was defined by Haralick et al.^36^. Eight sensitive texture parameters were selected to extract texture information from ZY-3 remote sensing images: mean, variance, uniformity, contrast, dissimilarity, entropy, second moment and correlation. The texture features change with the size, direction and step of the window. The most important issue for efficiently acquiring texture information is to use an appropriate computation window, because texture information will be lost in a too small window, while it will face excessive computation and storage pressure in a too large window. In this study, different windows (3*3, 5*5, and 7*7) are exploited to extract texture information, and the best computation window will be finally selected for the concluding tree species recognition^37^.

#### Cloud model

The cloud model was proposed by Li Deyi in 1995. The probability density function is applied to the cloud model to capture the uncertainty in the membership degree, which features fuzziness and randomness. Cloud model reflect the quantitative features of qualitative concepts by the digital characteristics of *expect* (*Ex*), *entropy* (*En*), and *hyper-Entropy* (*He*). *Ex* represents the most typical sample of a qualitative conceptual quantification. *En* reflects the uncertainty of the qualitative concepts; the greater *En* is, the more macroscopic the concept, and the greater the fuzziness and randomness are, the more difficult the concept quantification. *He* reflects the uncertainty of the entropy^19^. In this study, *Ex* denotes the typical eigenvalues of each tree species. *En* denotes the uncertainty of a tree species, which is the dispersion of the remote sensing feature information that could be classified in the sample area. *He* denotes the uncertainty of *En*, and it reflects the cohesion, i.e., cloud thickness, of the pixel belonging to a certain tree species.

The cloud generator includes the forward and backward cloud generators, and the forward one is also named X conditional cloud generator, which is adaptable to the condition of the digital features *Ex*, *En*, *He* and the test sample are available. There are three steps in the tree species classification algorithm based on the cloud model. First, the cloud model of each tree species is generated by backward cloud generator. Second, the membership degree of the samples is calculated with the X conditional cloud generator. Finally, the tree species were recognized according to the maximum determination method^19^. In this study, a three-dimensional cloud model was constructed based on the one-dimensional cloud model, and the recognition accuracies were compared with those of the one-dimensional cloud models.

(1) Backward cloud generator

**Figure 3:**
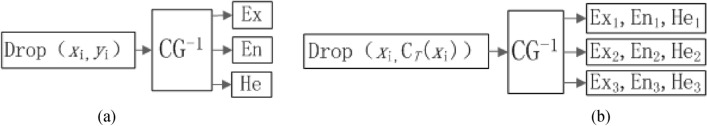
Backward cloud generator. (**a**) One-dimensional and (**b**) three-dimensional.

The sensitive indices or texture parameters of the modelling samples that represented the characteristic information of the tree species were input (${x}_{i}(i=\mathrm{1,2}....,n)$); then, the tree characteristic values of the cloud model were output (*Ex*, *En* and *He*), in which $Ex =\stackrel{\_\_}{X}=\frac{1}{n}{\sum }_{i=1}^{n}xi$, $En=\sqrt{\frac{\pi }{2}}\times \frac{1}{n}{\sum }_{i=1}^{n}|xi-\stackrel{\_\_}{X|}$, $He=\sqrt{{S}^{2}-E{n}^{2}}$. The one-dimensional and three-dimensional backward cloud generators are shown in Fig. 3a,b, respectively.

(2) X conditional cloud generator

The tree characteristic values of the cloud model (*Ex*, *En*, and *He*) and the corresponding sensitive indices or texture parameters of validation samples (${x}_{0}$) were input; then, the membership value for each tree species in each sample was output (${\mathrm{C}}_{T}\left({x}_{\mathrm{i}}\right)$), in which ${\varvec{C}}{\varvec{T}}({\varvec{X}}{\varvec{i}})={\varvec{exp}}[\frac{-({\varvec{X}}0-{\varvec{E}}{\varvec{X}}{)}^{2}}{2({\varvec{E}}{\varvec{n}}{\varvec{i}}\boldsymbol{^{\prime}}{)}^{2}}]$ ^38^.

(3) Maximum determination method

**Figure 4:**
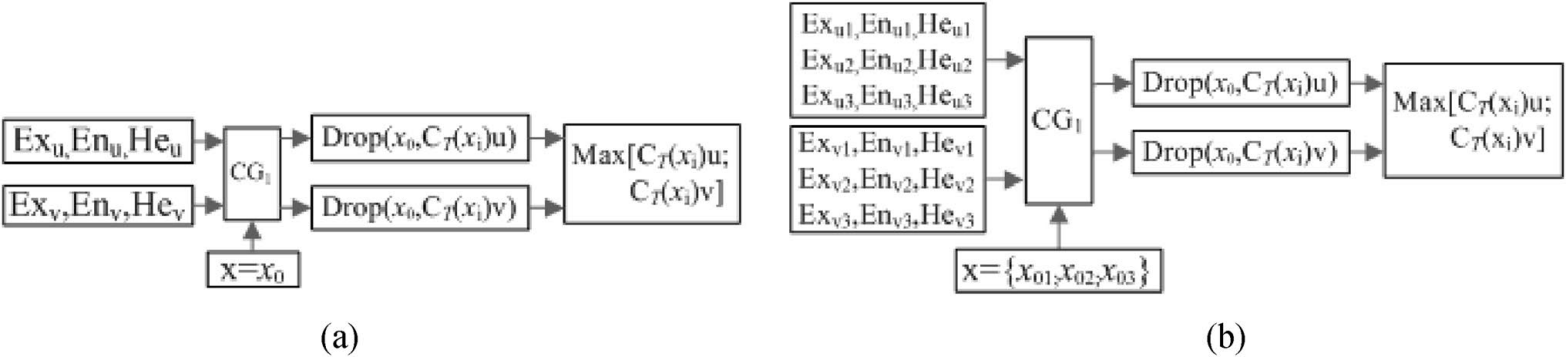
A schematic diagram of the $X$ conditional cloud generator and maximum determination method. (**a**) One-dimensional and (**b**) three-dimensional.

According to the maximum determination method, the membership values of the samples were calculated for every tree species^39^, and the tree species was recognized by selecting the maximum value. The principles of the one-dimensional and three-dimensional X conditional cloud generators and the maximum determination method are shown in Fig. 4a,b, respectively, in which u and v represent the tree species.

#### Support vector machine

Support vector machine (SVM) is a pattern recognition method based on statistical learning theory^40^. The basic idea is to map the data from the original feature space to a high-dimensional feature space through the kernel function. Then, the optimal hyperplane in the feature space is established to maximize the classification interval, and the unknown samples can be recognized on the hyperplane^41^. Currently, the commonly used kernel functions are the linear kernel function, polynomial kernel function, radial basis function (RBF) and sigmoid kernel function. Studies have shown that SVM classifiers constructed with radial kernel functions have better classification results^9,42^. A radial kernel function was chosen to construct the SVM classifier in this study.

## Results

### Sensitive spectral index

**Figure 5:**
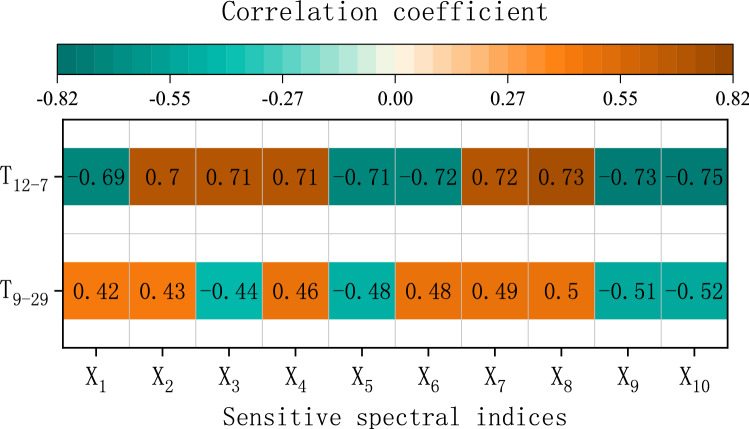
Correlation coefficients of sensitive spectral indices and tree species.

The correlation coefficients between the spectral indices and tree species were analysed, and 10 spectral indices with the highest correlation values at each phase were selected as sensitive spectral indices, as shown in Table 2. The correlations coefficients between the spectral index and tree species were divided into eight categories, i.e., 0.00 to ± 0.30, ± 0.30 to ± 0.50, ± 0.50 to ± 0.80, and ± 0.80 to ± 1.00, which indicating micro, real, significant and highly positive or negative correlations^43,44^, as shown in Fig. 5.

**Table 2:** Sensitive spectral indices.

	September 29	December 7
X_1_	*R*_*2*_ − *R*_*4*_	*R*_*4*_*/R*_*3*_
X_2_	(*R*_*1*_ − *R*_*4*_)*/*(*R*_*1*_*R*_*4*_)	(*R*_*4*_*/R*_*3*_)*/*(*R*_*4*_ − *R*_*3*_)
X_3_	(*R*_*4*_ − *R*_*2*_)*/*(*e*^*R4*^)	*lnR*_*3*_*/*(*R*_*3*_ − *R*_*4*_)
X_4_	(*R*_*3*_ − *R*_*4*_)*/*(*R*_*3*_*R*_*4*_)	*R*_*3*_*/R*_*4*_
X_5_	(*R*_*2*_*/R*_*4*_)*/*(*R*_*2*_ − *R*_*4*_)	*lnR*_*4*_*/*(*R*_*4*_ − *R*_*3*_)
X_6_	(*R*_*2*_*/R*_*4*_)*/*(*R*_*2*_ + *R*_*4*_)	*R*_*4*_ − *R*_*3*_
X_7_	(*R*_*3*_*/R*_*4*_)*/*(*R*_*3*_ + *R*_*4*_)	*R*_*3*_ − *R*_*4*_
X_8_	*R*_*2*_*/R*_*4*_	(*R*_*3*_ − *R*_*4*_)*/*(*e*^*R3*^)
X_9_	*R*_*4*_*/R*_*1*_	(*R*_*4*_ − *R*_*3*_)*/*(*e*^*R4*^)
X_10_	*R*_*4*_*/R*_*3*_	(*R*_*3*_*/R*_*4*_)*/*(*R*_*3*_ − *R*_*4*_)

X_1_ − X_10_ represents the categories of the 10 sensitive spectral indices. *R* represents the different band reflectance of the images.

The spectral indices ${\mathrm{X}}_{1}-{\mathrm{X}}_{7}$ on September 29 shown the real positively and negatively correlation with tree species, and ${\mathrm{X}}_{8}-{\mathrm{X}}_{10}$ on September 29 shown significant positive or negative correlation. The spectral indices ${\mathrm{X}}_{1}-{\mathrm{X}}_{10}$ on December 7 all shown significant positive and negative correlation with tree species. The correlation on December 7 was generally higher than that on September 29. The most dominant bands included in all sensitive spectral indices are band 4 on September 29 and band 3 and 4 on December 7, which indicates that the spectral indices constructed by band 3 and 4 shown the most robust correlation with the tree species.

### One-dimensional cloud model

#### Spectral features

**Figure 6:**
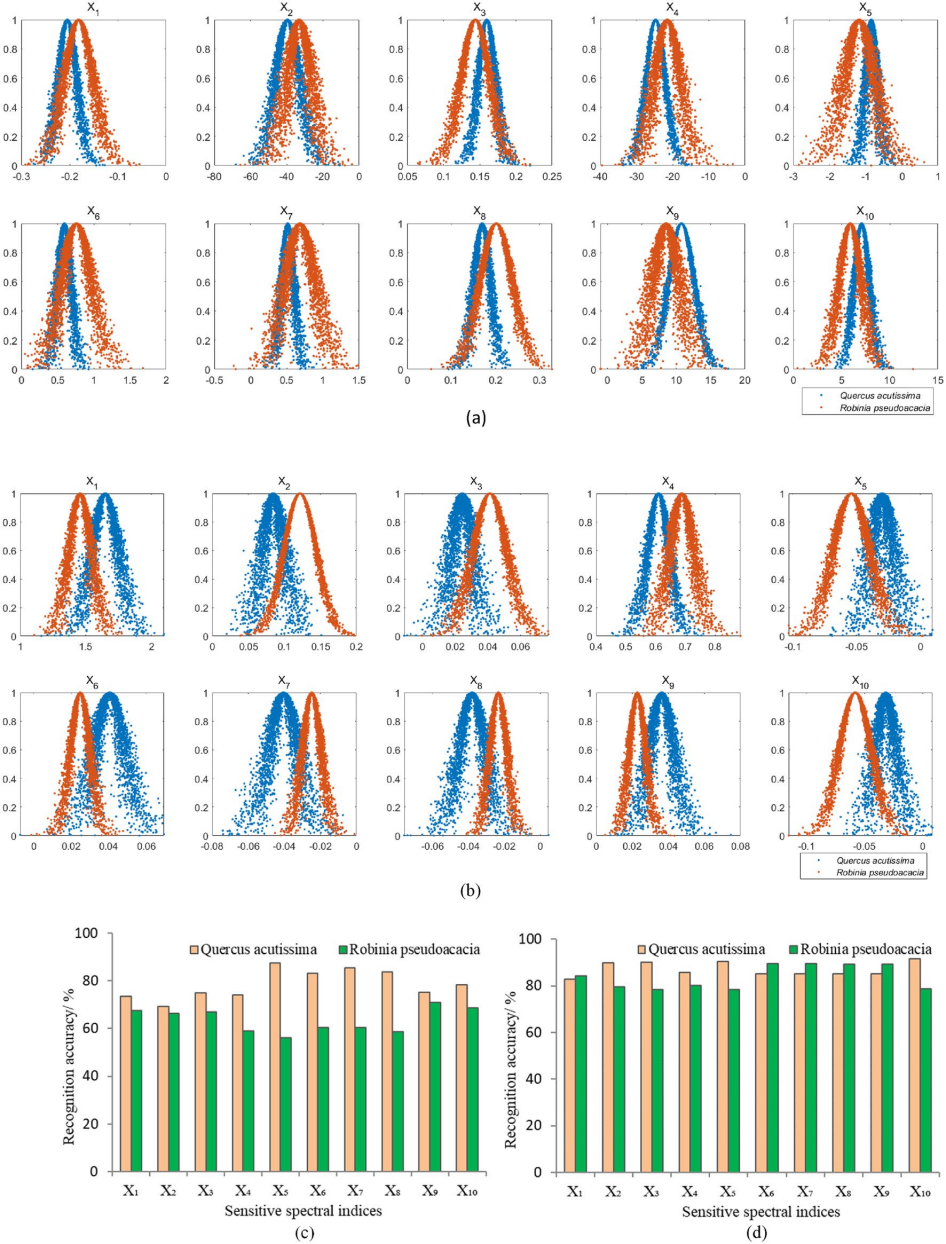
One-dimensional cloud model and recognition accuracies based on spectral characteristics. (**a**) One-dimensional cloud model constructed by spectral indices from September 29. (**b**) One-dimensional cloud model constructed by spectral indices from December 7. (**c**) Recognition accuracy of one-dimensional cloud model for both *Q. acutissima* and *R. pseudoacacia* in September 29. (**d**) Recognition accuracy of one-dimensional cloud model for both *Q. acutissima* and *R. pseudoacacia* in December 7. X_1_ − X_10_ represent the sensitive spectral indices.

The sensitive spectral indices of both *Q. acutissima* and *R. pseudoacacia* were used for obtaining the respective indices eigenvalues to construct the one-dimensional cloud models, as shown in Fig. 6a,b. The cloud model eigenvalues, *expect* (*Ex*), *entropy* (*En*), and *hyper-Entropy* (*He*), showed significant differences with sensitive spectral indices for different tree species. The shapes of the cloud droplet combinations for the cloud models of *Q. acutissima* and *R. pseudoacacia* are shown in Fig. 6a,b.

The recognition accuracy of the one-dimensional cloud model between different tree species has obvious variability, and the cloud models constructed with different sensitive spectral indices for one tree species also showed variable performance, as shown in Fig. 6c,d. On September 29, X_5_ had the highest accuracy (87.29%) for recognizing *Q. acutissima*, followed by X_7_ (85.41%) and X_8_ (83.65%), and the rest sensitive spectral indices were all higher than 69.18%. For *R. pseudoacacia*, X_9_ had the highest accuracy (71.02%), followed by X_10_ (68.66%) and X_1_ (67.57%), and the other sensitive spectral indices were all higher than 55.98%. On December 7, X_10_ had the highest accuracy (91.65%) for the recognition of *Q. acutissima*, followed by X_5_ (90.47%) and X_3_ (90.12%), and the other sensitive spectral indices were all higher than 82.82%. For *R. pseudoacacia*, X_6_ and X_7_ had the highest accuracies (89.49%), followed by X_9_ (89.31%), and the other sensitive spectral indices were all higher than 78.32%.

Generally, the average recognition accuracies on *Q. acutissima* and *R. pseudoacacia* were 78.46% and 63.50% on September 29, and 87.15% and 83.68% on December 7. We found that the recognition performance of the one-dimensional cloud model constructed by the sensitive spectral index of *Q. acutissima* was superior to that of *R. pseudoacacia*. Meanwhile, the recognition accuracy of December 7 spectral data derived from ZY-3 images was significantly higher than that of September 29 for both *Q. acutissima* and *R. pseudoacacia*.

#### Texture features

**Figure 7:**
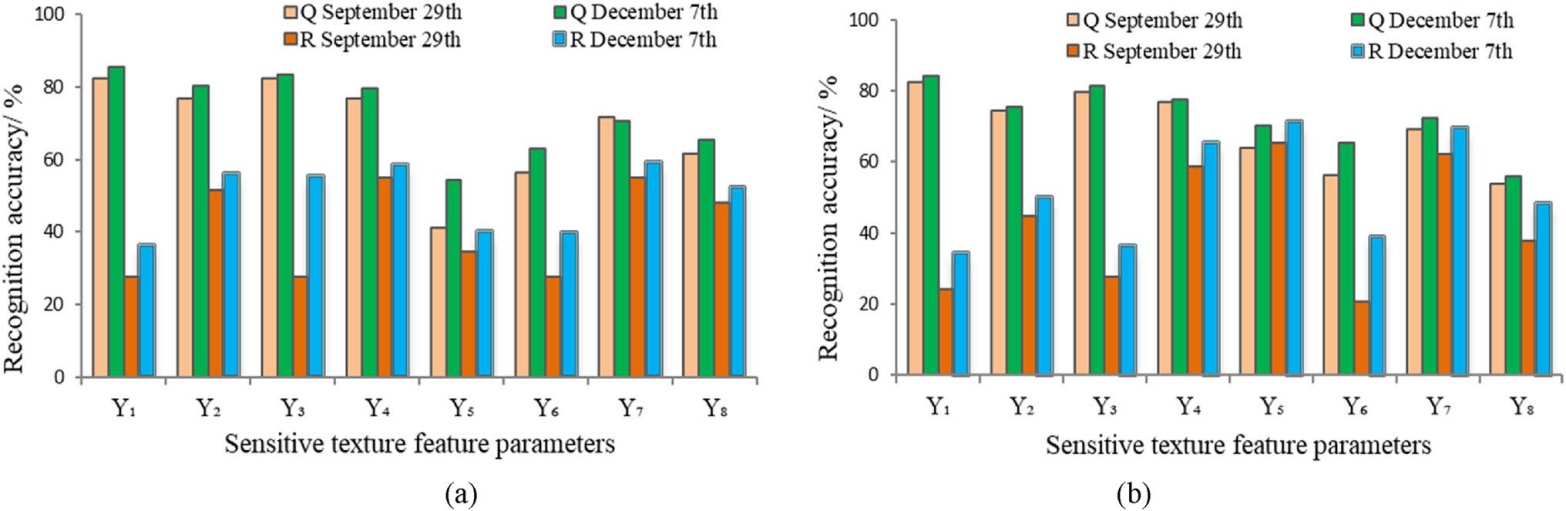
One-dimensional cloud model recognition accuracy based on texture features. (**a**) 3*3 window and (**b**) 5*5 window. Note: Y_1_ − Y_8_ represent the eight sensitive texture parameters of mean, variance, homogeneity, contrast, dissimilarity, entropy, second moment and correlation. Q and R represent *Q. acutissima* and *R. pseudoacacia*, respectively.

The recognition results on tree species based on texture features varied under different windows. *Q. acutissima* had the best recognition accuracy under 3*3 window, and *R. pseudoacacia* had the highest recognition accuracy under 5*5 window. Therefore, the optimal recognition accuracy of *Q. acutissima* under 3*3 window and that of *R. pseudoacacia* under 5*5 window were selected for the final accuracy analysis. The texture feature parameters Y_1_, Y_3_, and Y_4_ shown the highest recognition accuracy of 82.31%, 82.59% and 76.92% for *Q. acutissima* on September 29, while 85.71%, 83.56% and 79.82% on December 7, as shown in Fig. 7a. The texture feature parameters Y_5_, Y_7_, and Y_4_ shown the highest recognition accuracy of 65.52%, 62.07% and 58.62% for *R. pseudoacacia* on September 29, while 71.55%, 69.82% and 65.49% on December 7, as shown in Fig. 7b. The recognition accuracy on December 7 was higher than that on September 29. In which Y_1_ had the highest recognition accuracy of 85.71% for *Q. acutissima* and Y_5_ had the highest recognition accuracy of 71.55% for *R. pseudoacacia*. We found that the recognition accuracy of one-dimensional cloud models constructed by texture features was lower than that of spectral features for *Q. acutissima* and *R. pseudoacacia* on both September 29 and December 7.

### Three-dimensional cloud model

**Figure 8:**
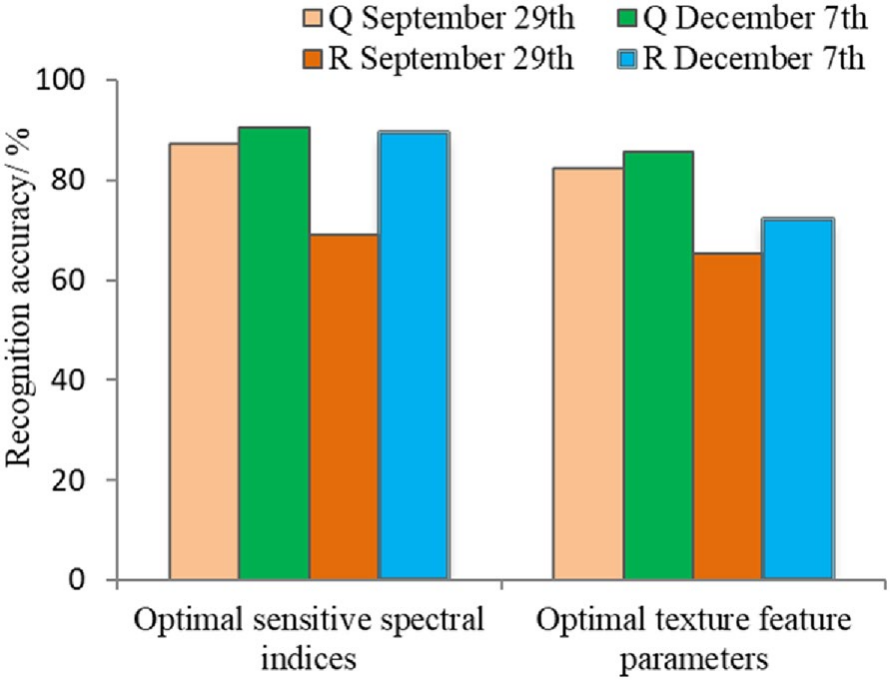
Recognition accuracy of the three-dimensional cloud model. Note: Q and R represent *Q. acutissima* and *R. pseudoacacia*, respectively. The optimal texture feature parameters for constructing *Q. acutissima* cloud model are extracted under 3*3 window, while those for *R. pseudoacacia* cloud model are under 5*5 window.

The three-dimensional cloud models were constructed by the optimal three sensitive spectral indices or the three optimal texture feature parameters selected by the recognition accuracy of the one-dimensional cloud model. The optimal sensitive spectral indices on September 29 were X_5_, X_7_ and X_8_ for *Q. acutissima* and X_1_, X_9_ and X_10_ for *R. pseudoacacia*. The optimal sensitive spectral indices on December 7 were X_3_, X_5_ and X_10_ for *Q. acutissima* and X_6_, X_7_ and X_9_ for *R. pseudoacacia*. The optimal texture feature parameters of the two temporal phases were Y_1_ (mean), Y_3_ (homogeneity) and Y_4_ (contrast) for *Q. acutissima* and Y_4_ (contrast), Y_5_ (dissimilarity) and Y_7_ (second moment) for *R. pseudoacacia*, as shown in Fig. 5. Figure 8 shows that the overall recognition accuracies for the two tree species on September 29 and December 7 were 78.25% and 89.89% based on the spectral features and 73.75% and 78.92% based on the texture features, respectively.

In summary, the remote sensing recognition accuracies of the three-dimensional cloud models for *Q. acutissima* and *R. pseudoacacia* showed that December 7 was superior to September 29 and the spectral features were superior to the texture features. Moreover, the recognition accuracy of the three-dimensional cloud model on *Q. acutissima* was generally higher than that of *R. pseudoacacia*, and the three-dimensional cloud model was not found to have significantly improved performance over the one-dimensional cloud model.

### Support vector machine

The spectral features on December 7 showed a high accuracy and used to construct the SVM recognition models as contrast with cloud model, in which the one-dimensional and three-dimensional SVM (SVM_1_ and SVM_3_) were constructed separately by 10 single sensitive spectral indices and 3 optimal sensitive spectral indices. Modelling, accuracy verification and parameter optimization were performed to determine that C-support vector classification (C-SVC) as the SVM type and RBF as the SVM kernel function. In addition, the SVM model parameters constructed by different spectral index variables were kept consistent to ensure the SVM recognition results were relatively comparable, as shown in Table 3.

**Table 3:** The model parameters of the SVM.

Tree species	Degree	Gamma	Coef0	Epsilon	C	Nu	Shrinking	p
*Q. acutissima*	3	0.5	0.001	0.001	1	0.5	1	1
*R. pseudoacacia*	3	0.5	0.001	0.001	1	0.5	1	1

**Figure 9:**
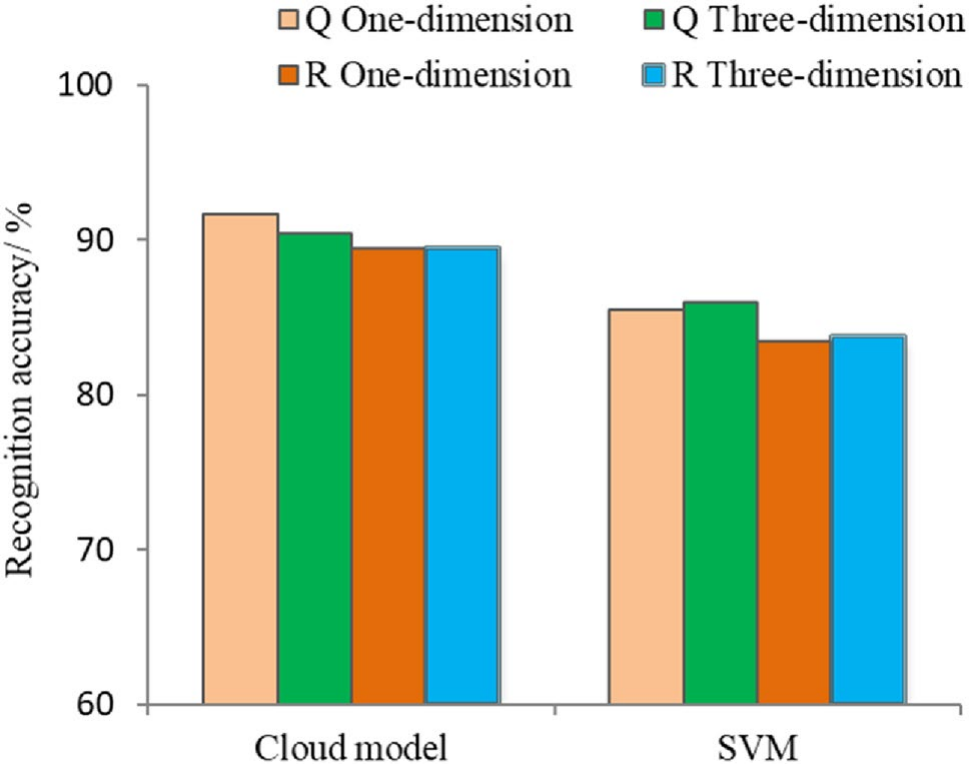
Recognition accuracy comparison between the cloud model and SVM. Note: Q and R represent *Q. acutissima* and *R. pseudoacacia*, respectively. One-dimension and three-dimension represent the different dimensional recognition model.

The recognition accuracies of the cloud models and SVM are shown in Fig. 9. SVM_1_ and SVM_3_ had recognition accuracies of 85.45% and 85.94% for *Q. acutissima* and 83.51% and 83.78% for *R. pseudoacacia*, respectively, and non significant differences in the recognition results were found between SVM_3_ and SVM_1_. The recognition performance of the cloud model and SVM was compared and found that the optimal recognition accuracy of SVM_3_ for the two tree species is 84.86%, which is lower than the 89.98% from three-dimensional cloud model. Meanwhile, the optimal recognition accuracy of SVM_1_ for the two tree species is 84.48%, which is lower than the 90.57% of the one-dimensional cloud model. The average recognition accuracy of 77.72% from 10 SVM_1_ models constructed by the 10 sensitive spectral indices was also lower than that of 85.42% from 10 one-dimensional cloud models. The results indicate that the cloud model outperforms the support vector machine for the recognition of *Q. acutissima* and *R. pseudoacacia*.

## Discussion

Spectral and texture features from ZiYuan-3 satellite were put into one-dimensional or multi-dimensional cloud model and support vector machine model for the remote sensing recognition of *Q. acutissima* and *R. pseudoacacia* on Mount Tai. The recognition accuracies of the tree species were analysed to find the discriminative image features and optimal recognition model in this study.

Tree species recognition performance of the cloud model constructed with both sensitive spectral indices and texture feature parameters was found to be superior on December 7 than on September 29. The explanation for the difference in recognition performance is speculated to be, on the one hand, the rough discrimination of the canopy layer due to the luxurious growth of the broadleaf species. On the other hand, the leaf color of deciduous species was undergoing a regular change in late autumn^45^, and the mixture of green and yellow leaf colors contributed to the confusion of the spectral features from specific tree species. In contrast, on December 7, *Q. acutissima* and *R. pseudoacacia* are experiencing the defoliation stage in early winter, when the leaves were fully discolored and partially defoliated. Then the trunk morphological differences exposed in this period provide characteristic spectral information, and the differences in leaf surface morphology, moisture and chlorophyll content of the tree species at different defoliation stages all contribute to the variations in spectral information.

The recognition accuracy by multispectral information on *Q. acutissima* was generally higher than that on *R. pseudoacacia*, which may be explained by the differences in crown width, tree height and leaf tyle. Moreover, altitude, gradient, slope direction, and tree age may also induce differences in the spectral reflectance and texture features of tree species. Accordingly, the investigation of the morphology, planting environment, and age of tree species is regarded as one of the critical issues for further development of forest survey and monitoring with the remote sensing technology.

The performance improvement of remote sensing recognition in winter over autumn was more significant for *Q. acutissima* than for *R. pseudoacacia*. For example, the recognition accuracy of the cloud model constructed with five sensitive spectral indices X_4_ − X_8_ from the September 29 image was only about 60% for *R. pseudoacacia* (Fig. 6c), while the recognition accuracy from the December 7 image improved up to 90% (Fig. 6d). This indicates that the recognition accuracy of December 7 is improved by about 30% over that of September 29 for *R. pseudoacacia*, while *Q. acutissima* was found only improved by about 10%. The recognition performance of the cloud model constructed by the sensitive spectral indices from September 29 for *Q. acutissima* were all superior to that of *R. pseudoacacia*, while the recognition accuracy gap between the two species from December 7 was narrowed considerably, and even *R. pseudoacacia* surpassed *Q. acutissima*. Consequently, the recognition performance of multispectral information of *R. pseudoacaci* in winter was revealed to be significantly improved than that in autumn, and the explanation for this phenomenon was explored in this study. The average tree height is 6.93 m and the average diameter at breast height is 12.9 cm, according to Hao et al*.*^45^, who surveyed 238 *Q. acutissima* individuals in the field on Mount Tai. Mi et al*.*^46^ concluded that the average tree height of *Q. acutissima* on Mount Tai is 8 m, diameter at breast height is 9.1 cm, and crown density is 0.5, while the average tree height of *R. pseudoacaci* is 8–9 m, diameter at breast height is 15 cm, and crown density is 0.8–0.9. Therefore, as the trunk is exposed after the leaves fall in winter, the larger size and canopy density as well as the curved trunk morphology of *R. pseudoacaci* make it more easily captured by remote sensing technology, and thus the recognition accuracy for *R. pseudoacaci* in winter was significantly improved.

Spectral features were found to have a higher recognition accuracy than textural features for recognizing on *Q. acutissima* and *R. pseudoacacia.* A potential explanation for this conclusion could be that the tree species features revealed by the spectral indices are a collection of multiple features such as canopy water content and leaf chlorophyll content, while the texture feature indices only mainly revealed information on the orthometric geometry of the tree species.

Researchers have evaluated the performance of the existing tree recognition algorithms for recognizing tree species. The minimum distance and maximum likelihood methods were the traditional algorithms in remote sensing recognition. These methods characterised by mature application, simple implementation, a small amount of computation and fast speeds, but the disadvantage is that the recognition accuracy was insufficient, and only the samples with obvious features could be correctly recognized. SVM recently have been widely used for the accurate recognition of forest species and shown higher recognition performance than other methods^12^. Clouds are a new and readily visualizable concept of uncertainty. Cloud models are an effective tool for converting qualitative concepts into quantitative formulations and are capable of capturing fuzzy and stochastic uncertainty with simple numerical features. Cloud model have the advantage of simplicity of operation and not needing extensive model computation. In this research, the cloud model has also been proved outperforming the support vector machine for the recognition on *Q. acutissima* and *R. pseudoacacia*. The recognition accuracy of the multidimensional model does not show a superior advantage over the one-dimensional model, both for cloud model and support vector machine. The one-dimensional cloud model was simple and required fewer spectral indices and calculation procedures, which can be used as the priority cloud model. The operation simplicity and performance superiority of cloud models will bring an extensive prospect for its application in remote sensing recognition on tree species.

Sample data availability of tree species is essential no matter which classification algorithm is applied. Both the quantity and quality of sample data should be greatly expanded in the future research on the tree species recognition of Mount Tai with remote sensing technology, which is the foundation for better recognition accuracy. The tree species involved in this study are relatively limited and should be widely expanded in future studies, providing a comprehensive technical support for forest resources survey and monitoring of Mount Tai.

## Conclusions

The multispectral and texture features of the remote sensing images from the ZiYuan-3 (ZY-3) satellite at two phenological phases of autumn and winter (September 29th and December 7th) were selected for constructing and optimizing sensitive spectral indices and texture indices. Multidimensional cloud model and support vector machine model were constructed by the screened spectral and texture indices for remote sensing recognition of *Q. acutissima* and *R. pseudoacacia* on Mount Tai. The results showed that, the correlation intensities of the constructed spectral indices with tree species were preferable in winter than in autumn. The spectral indices constructed by band 4 showed the superior correlation compared with other bands, both in the autumn and winter time phases. The optimal sensitive texture parameters for both time phases were mean, homogeneity and contrast for *Q. acutissima* and contrast, dissimilarity and second moment for *R. pseudoacacia*. The texture parameters Y_1_ (mean) have the highest recognition accuracy of 85.71% for *Q. acutissima* and Y_5_ (dissimilarity) have the highest recognition accuracy of 71.55% for *R. pseudoacacia*. The recognition performance of the both one-dimensional and three-dimensional cloud model constructed by the sensitive spectral index of *Q. acutissima* was superior to that of *R. pseudoacacia*. Meanwhile, the recognition accuracy of December 7 spectral data derived from ZY-3 images was significantly higher than that of September 29 for both *Q. acutissima* and *R. pseudoacacia*. Spectral features were found to have a higher recognition accuracy than textural features for recognizing on *Q. acutissima* and *R. pseudoacacia,* and winter showing superior recognition accuracy than autumn, especially for *Q. acutissima*. The recognition accuracy of the multidimensional cloud model (89.98%) does not show a superior advantage over the one-dimensional cloud model (90.57%). The one-dimensional cloud model was simple and required fewer calculations, which can be used as the priority cloud model. Non significant differences in the recognition results were found between one-dimensional and three-dimensional support vector machine model. The highest recognition accuracy derived from SVM_3_ was 84.86%, which was lower than the cloud model (89.98%) in the same dimension. The cloud model outperforms the support vector machine for the recognition of *Q. acutissima* and *R. pseudoacacia*. The research results are expected to provide technical support for the precise recognition and forestry management on Mount Tai.

## Data Availability

The datasets generated and/or analysed during the current study are not publicly available due the confidentiality agreements but are available from the corresponding author on reasonable request.
